# Addressing the COVID-19 emergency during the ongoing political and economic crisis in Fragile Lebanon: a call to action

**DOI:** 10.1186/s13031-021-00403-3

**Published:** 2021-09-03

**Authors:** Ludmila Lobkowicz, Julien Lahoud, Ibrahim Bou-Orm

**Affiliations:** 1 Order of Malta Lebanon, Beirut, Lebanon; 2 Institute for Global Health and Development, Queen Margaret University, Edinburgh, EH21 6UU UK

**Keywords:** COVID-19, Humanitarian crisis, Vaccination

## Abstract

This Letter to the Editor aims to reflect on the current challenges to increase the coverage of COVID-19 vaccination in the fragile and conflict-affected setting of Lebanon, which is currently experiencing one of the biggest economic crises globally as well as a recent surge in COVID-19 cases. Addressing the supply- and demand-related factors affecting vaccination would increase COVID-19 vaccine coverage and prevent the complete collapse of an already overwhelmed Lebanese health care system.

## Main Text

Lebanon is currently enduring a very severe financial and economic crisis [1]. This started late 2019 and exacerbated in 2020 with the ongoing COVID-19 pandemic and the enormous destruction of major parts of the Capital city of Beirut due to the Beirut Port explosion in August 2020 [2]. In 2021, the economy and monetary crisis has reached a critical level which pushed families and communities to poverty and threatened the activity of all sectors including the health sector [2]. For instance, by the time we are writing this letter, the local currency has lost more than 90% of its value [2]. The downstream effects of this currency devaluation, such as skyrocketing prices and shortages of basic supplies, including medication and medical equipment, most of which are imported, along with unreliable power conditions due to an ongoing fuel crisis, are pushing the health care system to the verge of collapse [2]. In addition, the political system struggles with another never-ending deadlock which blocks the creation of a government to initiate a set of reforms that the international community requires as a pre-requisite for channeling aid and support. The reasons behind this challenging situation include a political system, which is based on power-sharing between different religious denominations that distorted the relationship between the state and its “citizens” to clientelist approaches [3]. The country witnessed a civil war between 1975 and 1990 given its critical geopolitical position in the Middle East as well as several political deadlocks and security problems that exacerbated after 2005, the year when the Syrian troops withdrew from Lebanon [1, 2]. The situation worsened with the ongoing Syrian crisis, that started in 2011 and has caused a Syrian refugee flow as well as spill-over security issues in Lebanon [2]. Currently alongside its population of about 5,3 million, the Lebanon hosts an estimate of 1.5 million refugees who have fled the conflict in Syria, of whom 865,531 are registered with the United Nations High Commissioner for Refugees (UNHCR)and 27,803 are Palestinian refugees from Syria registered with United Nations Relief and Work Agency (UNRWA) [4, 5]. In addition, there are 479,537 Palestine refugees in Lebanon registered with UNRWA [6].

Within this turbulent political economy, Lebanon is now under the threat of a new wave of COVID-19 with the 100% increase of COVID-19 cases over the past 14 days, adding up to about 1502 new daily COVID-19 cases on the 27th of July 2021 [7, 8] This increase in cases is most likely caused by the spread of the B.1.617.2 (delta) variant, which has globally been described to be highly transmissive and first reported to be circulating in Lebanon on the 2nd of July 2021 [9, 10]. In parallel, the Lebanese Ministry of Public Health (MoPH) reported that only 1,098,457 people (16%) were vaccinated with a single dose of the COVID-19 vaccine and 789,054 people (~ 12%) were vaccinated with two doses by the 28th of July 2021 [8, 11] three observations are noted: (1) the low percentage of coverage which is far from the threshold for herd immunity; (2) the flattening of the vaccination curve as of the second week of July especially for the uptake of the first dose; (3) potential uptake inequity among communities. For instance, although the MoPH’s initial national vaccination plan included all people currently residing in Lebanon, the distribution of to date administered vaccinations per nationality displays that about 93% (n = 1,746,387) of vaccinations were administered to Lebanese, 1.8% (n = 33,331) to Palestinians and 1.4% to Syrians (n = 26,015), reflecting an unbalanced distribution of vaccines administration per nationality, given that only registered Syrian refugees made up 13% of all the people residing in Lebanon in 2020 [8, 12]. Reasons for this inequitable and low vaccination coverage might be related to the quantity of available vaccines in the country, but mainly to challenges in the delivery of the vaccination service and the related policies. For instance, the MoPH had to cancel many vaccination marathons due to the inability to operate centers given some power and internet issues [13]. Moreover, there is no clear vision on the constant change in priority lists for vaccination, and health actors are not addressing community perceptions about the vaccination to increase the uptake of the vaccine.

Given that vaccination is the most cost-effective strategy to control the pandemic, Lebanon has to speed up the vaccination process as the health sector cannot afford any additional shock from the COVID-19 pandemic and does not have the same capacities that were available to address the previous shock in early 2021 [14]. This letter is a call for all health actors in Lebanon and globally to support Lebanon in scaling up the vaccination process from vaccines’ supply to ensuring rapid distribution of vaccines in well-equipped and supported centers and overcoming community-related obstacles for the uptake of this preventive measure.

Practically, the three most urgent recommendations are to (1) advocate for vaccine donations from high-income countries given the insufficient number of doses allocated for Lebanon by the COVAX alliance and the limited financial capacity to buy more doses [15]; (2) secure fuel supply to keep vaccination centres open and enable potential outreach activities especially in distal/underserved areas; (3) conduct awareness campaigns especially among young generations to overcome the vaccine hesitancy and to stress on the importance of vaccination to protect from COVID-19 including related hospitalizations and deaths [16].

## Data Availability

Not applicable.

## Abbreviations

COVID: Coronavirus disease
MoPH: Ministry of Public Health
UNHCR: United Nations High Commissioner for Refugees
UNRWA: United Nations Relief and Work Agency
WHO: World Health Organization
